# Comparison of ultrasonography-based masticatory muscle thickness between temporomandibular disorders bruxers and temporomandibular disorders non-bruxers

**DOI:** 10.1038/s41598-024-57696-6

**Published:** 2024-03-22

**Authors:** Yeon-Hee Lee, Yang-Hyun Chun, Hyungkyu Bae, Jung-Woo Lee, Hee-Jin Kim

**Affiliations:** 1 Department of Orofacial Pain and Oral Medicine, Kyung Hee University, Kyung Hee University Dental Hospital, #613 Hoegi-dong, Dongdaemun-gu, Seoul, 02447 South Korea; 2 Division in Anatomy and Developmental Biology, Department of Oral Biology, BK21 FOUR Project, Human Identification Research Institute, Yonsei University College of Dentistry, Seoul, South Korea; 3 Department of Oral and Maxillofacial Surgery, School of Dentistry, Kyung Hee University, Seoul, 02447 South Korea

**Keywords:** Ultrasonography, Temporomandibular disorder, Masseter muscle, Temporalis muscle, Thickness, Bruxism, Medical research, Risk factors, Signs and symptoms

## Abstract

To compare masticatory muscle thickness in patients with temporomandibular disorders (TMDs) during rest and clenching, and by body position, using ultrasonography. This prospective study included 96 patients with TMD (67 females, 29 males; mean age: 40.41 ± 17.88 years): group 1, comprising 66 patients with TMD without bruxism (TMD_nonbruxer), and group 2, comprising 30 patients with concurrent TMD and bruxism (TMD_bruxer). In patients with TMD, bruxism was correlated with the presence of tinnitus, muscle stiffness, sleep problems, psychological stress, and restricted mouth opening. The masseter muscle significantly thickened during clenching (11.16 ± 3.03 mm vs 14.04 ± 3.47 mm, *p* < 0.001), whereas the temporalis muscle showed no significant increase in thickness from resting to clenching in an upright position (7.91 ± 1.98 vs 8.39 ± 2.08, *p* = 0.103). Similarly, during clenching in the supine position, the masseter muscle was significantly thicker compared with rest (11.24 ± 2.42 vs 13.49 ± 3.09, *p* < 0.001), but no significant difference was observed in temporal muscle thickness (8.21 ± 2.16 vs 8.43 ± 1.94, *p* = 0.464). In comparison between two groups, the average thickness of the masseter muscle was greater among TMD_bruxers than among TMD_nonbruxers in both the upright and supine positions (all *p* < 0.05). In the generalized lineal model, female sex (B = − 1.018, 95% confidence interval [CI] − 1.855 to − 0.181, *p* = 0.017) and bruxism (B = 0.868, 95% CI 0.567 to 1.169, *p* = 0.048) significantly predicted changes in masseter muscle thickness. Female sex (B = − 0.201, 95% CI − 0.299 to − 0.103, *p* = 0.011), increased age (B = − 0.003, 95% CI − 0.005 to 0.000, *p* = 0.038), and muscle stiffness (B = − 1.373, 95% CI − 2.369 to − 0.376, *p* = 0.007) were linked to decreased temporal muscle thickness. Comparing TMD nonbruxer and bruxer muscle thicknesses in upright and supine positions revealed significant increased thickness in the masseter muscle during clenching but not in the temporalis muscle. Masseter muscle thickness varied significantly by sex, body position, and resting/clenching, notably influenced by bruxism. These findings emphasize the relevance of these factors in clinical examinations of patients with TMD.

## Introduction

Temporomandibular disorders (TMDs) is a broad term encompassing structural abnormalities, disabilities, or pain affecting the temporomandibular joint (TMJ) and related muscular and bony components. Symptoms vary widely among patients, including tinnitus, ear pain, psychological issues, sleep disturbances, and TMJ-related discomfort or pain^1,2^. The evidence-based diagnostic criteria for TMD (DC/TMD), globally adopted in 2014, encompass 12 categories, including arthralgia, myalgia, local muscle pain, myofascial pain, referred myofascial pain, four types of disc displacement disorders, and degenerative joint disease^3^. These criteria are prevalent within the TMD spectrum, classifying TMD into arthrogenous, myogenous, and mixed types based on pain origin^4^.

TMD etiology is multifactorial, involving factors such as bruxism, occlusal abnormalities, orthopedic instability, microtrauma, macrotrauma, and female hormonal changes^5,6^. Psychological stress, mental pressure, anxiety, depression, and somatization are additional contributing factors^7^. TMD is common, with a prevalence ranging 5%–12%^3^, particularly among younger individuals in their 20 s and 40 s^8^. Women experience TMD more frequently than men, with at least double the prevalence rate, and they tend to seek treatment for these conditions more often than men^9^.

Bruxism involves repetitive jaw muscle action—clenching or grinding teeth, and bracing or thrusting the mandible^10^. This may lead to varying thicknesses in the primary muscles responsible for these movements—the masseter and temporalis. Bruxism is a prevalent condition affecting 8%–31% of the population, irrespective of gender^11^. Awake bruxism affects approximately 22%–31% of adults, whereas approximately 13% exhibit sleep bruxism (SB)^11,12^. In adults, while not classified as a distinct disorder, bruxism is a potential risk factor for TMD^13^, increasing its likelihood. Patients with SB have higher risks of myofascial pain (odds ratio [OR], 5.93) and TMJ arthralgia (OR, 2.34)^14^. Given the disparity in etiology and pathogenesis between SB and awake bruxism, clinical differentiation poses a challenge for both clinicians and patients^15,16^. Consequently, has occasionally been regarded and investigated as a single entity^17–19^.

The etiology of bruxism, akin to TMD, involves multiple factors. Biological factors, including neurotransmitters (dopamine) and cortical arousal, constitute one group^20^. Psychological factors, such as stress susceptibility, personality traits, and anxiety, form another^21^. Research indicates higher stress and anxiety levels in both adult and child bruxism sufferers than in control groups^22^, indicating potential mental health implications. Exogenous factors, including substances such as nicotine, caffeine, alcohol, and drugs, are also implicated in causing bruxism^23^. Additionally, associations between bruxism and systemic disorders such as thyroid disease, digestive disorders, sleep disturbances, and cardiovascular diseases have been observed^24^. Bruxism may act as a trigger for TMD or share underlying developmental mechanisms.

Efforts have been made to use high-frequency ultrasonography for TMD diagnosis, effectively visualizing healthy muscles and joints and identifying related conditions^25^. Ultrasound imaging provides a real-time visualization of both normal and pathological muscle tissues with efficacy^26^, making it a convenient, noninvasive technique. Traditionally, imaging the TMJ and its surrounding muscles posed challenges. TMJ bony structure assessment relied on plain radiography, cone-beam computed tomography (CBCT), CT, and bone scintigraphy. Meanwhile, evaluating masticatory muscles was limited to magnetic resonance imaging (MRI) and ultrasonography^6,8,27^. Currently, ultrasonography stands as an accurate method for in vivo measurement of masticatory muscle thickness^28^. Raadsheer et al. found a strong correlation between muscle thickness measured via MRI and ultrasonography^29^. As of now, the use of ultrasonography for diagnosing TMD is not supported by the DC/TMD, which only mentions MRI for confirming soft-tissue abnormalities^3^. Moreover, studies investigating the use of ultrasound in assessing orofacial muscles affected by bruxism in patients with TMD are limited. These ultrasound studies focused on the masseter muscle among masticatory muscles: patients with myofascial pain and bruxism had significantly thinner and harder masseter muscle than healthy controls^30^, and in TMD patients with bruxism, exercise treatment did not contribute to reducing the masseter muscle thickness^31^.

This study explored ultrasonography-based alterations in masseter and temporalis muscle thicknesses concerning body positioning and occlusal resting/clenching states. In previous electromyographic (EMG) studies, the masseter muscle exhibited a more active role than the temporalis muscle during maximum clenching or mastication^32,33^. In healthy individuals, the masseter muscle thickness demonstrated a significant increase in contraction compared to at rest, while the temporalis muscle did not^34^. Ultrasound-based investigation is needed to assess changes in the masseter and temporalis muscles in patients with TMD under these conditions. Furthermore, in the TMJ and masticatory muscle testing protocol according to DC/TMD, patients are encouraged to assume a comfortably upright sitting position, while the examiner stands^35^. However, in clinical practice, depending on the various clinician, patients, and surrounding factors, the patient may be evaluated in a supine position on the dental unit chair. Nevertheless, no study has investigated muscle thickness or condition according to posture in TMD patients.

The study hypothesized that during tooth clenching, masseter muscle thickness would increase compared with that in the resting state, possibly further increasing with bruxism presence. Conversely, temporalis muscle thickness might not significantly change because of these factors. Additionally, this hypothesis suggested that body position (upright or supine) might not induce muscle thickness alterations. Variations in these muscles could impact TMD-related clinical factors. To test these hypotheses, we compared clinical characteristics and muscle thickness among patients with TMD based on posture and resting/clenching conditions.

## Methods

The research protocol for this study adhered to the principles of the Declaration of Helsinki and received approval from the Institutional Review Board of Kyung Hee University Dental Hospital, Seoul, South Korea (KHD IRB, IRB No-KH-DT22015). Informed consent was obtained from all participants.

### Participants

Ninety-six patients with TMD (67 females, 29 males; mean age, 40.41 ± 17.88 years) participated in this study at the Department of Orofacial Pain and Oral Medicine of the Kyung Hee University Dental Hospital (Seoul, South Korea) between June 2022 and March 2023. All patients underwent thorough examination and diagnosis by two TMD specialists with > 8 years of experience, using the DC/TMD examination. Additionally, patients completed comprehensive questionnaires, including the DC/TMD symptom questionnaire, DC/TMD demographics, and an oral behavior checklist. For the sample size calculation, we used the G*Power software (latest ver. 3.1.9.7; Heinrich-Heine-Universität Düsseldorf, Düsseldorf, Germany); 46 subjects (23 subjects per group, actual power = 0.95) were derived with an actual target of at least 30 per group as suitable for statistical analysis, and finally a total of 96 participants were recruited.

The patients were divided into two groups based on the presence and absence of bruxism: group 1 comprised 66 patients with TMD without bruxism (TMD_nonbruxers) (47 females, 71.2%; 41.27 ± 17.92 years) and group 2 comprised 30 patients with TMD concurrent bruxism (TMD_bruxers) (20 female, 66.7%; 38.51 ± 17.95 years). The age and sex distributions were not significantly different between the two TMD groups with and without bruxism (TMD_non-bruxers vs. TMD_bruxers) (all *p* > 0.05).

Participant selection was based on a standardized clinical examination. The inclusion criteria were as follows: all participants underwent a physical examination according to the DC/TMD^3^ and were aged ≥ 18 years. Consequently, individuals diagnosed with arthrogenous and/or myogenous TMD were included in the study. The exclusion criteria were: (1) age < 18 years, (2) history of facial tumor or surgery, (3) local facial infection, (4) systemic inflammatory connective tissue disease, (5) other systemic musculoskeletal disorders (fibromyalgia, rheumatoid arthritis, inflammatory joint disease), (6) neurologic impairment or diseases (stroke, tumor, epilepsy), (7) psychiatric disorders, (8) pregnancy, and (9) inability to provide informed consent.

### Study design

#### Clinical evaluation

(1) Characteristics of TMD pain

The duration of pain due to TMD has been reported in months. When the symptom duration was > 6 months, it was classified as TMD chronicity^4^. TMD pain was scored by the patients on a visual analog scale (VAS) ranging from 0 (no pain at all) to 10 (worst pain imaginable). The presence of preauricular pain was noted when the patient reported pain in or around the ear along with TMD pain^36^.

(2) Evaluation of bruxism

Bruxism is a movement disorder characterized by grinding and/or clenching of teeth, which may occur during wakefulness or sleep. In our assessment, patients were identified as having bruxism if they self-reported bruxism, indicating a positive response to two or more questions, including the initial inquiry.

Questionnaire for detecting bruxer^37^:

1. Has anyone observed or heard your teeth grinding at night?
2. Do you experience jaw fatigue or soreness upon waking up in the morning?
3. Do you notice any soreness in your teeth or gums upon waking up in the morning?
4. Do you experience temporal headaches upon waking up in the morning?
5. Are you aware of tooth grinding during the day?
6. Do you experience episodes of tooth clenching during the day?

These questions constitute the questionnaire used to identify potential bruxism, aiming to capture various symptoms and manifestations associated with tooth grinding and clenching throughout the day and night. Thus, in this study, bruxism encompasses probable bruxism and refers to both awake bruxism and/or sleep bruxism^12^.

(3) Diagnosis of TMD and TMD subgroups

TMD was diagnosed according to DC/TMD^3^. It can be broadly categorized into arthrogenous and myogenous types, depending on the origin and pattern of pain^4^. The three TMD subgroups consisted of arthrogenous TMD, myogenous TMD, and mixed TMD, which presents elements of both. Arthrogenic TMD includes conditions such as arthralgia, arthritis, osteoarthritis, and articular disc displacement. Myogenous TMD comprises local myalgia, myofascial pain, and myofascial pain upon referral. Mixed TMD refer to the coexistence of arthrogenous and myogenous TMD.

(4) Chief complaints of TMD patients

The chief complaints of TMD patients were identified in five categories: TMJ noise, TMD pain, limited mouth opening, muscle stiffness, and occlusal dysesthesia. Chief complaints were organized into one or two or more multiples based on patient reports. TMJ noise was considered present when clicking, fine and/or coarse crepitus, and popping noises in the TMJ occurred during mandibular movement^38^. TMD pain encompassed pain around the TMJ, muscles, ears, and temple areas^39^. Limited mouth opening referred to a situation where comfortable mouth opening was < 35 mm^40^, leading to difficulty or discomfort when opening the mouth. Muscle stiffness in the masticatory muscles was referred to as the sensation of tightness or pain in the muscles^41^. Occlusal dysesthesia described the perpetual perception of disturbing or unpleasant tooth contacts^42^.

(5) Contributing factors or comorbidities for TMD

The presence of psychological stress was evaluated using a dichotomous question: “Have you encountered any mental stress or psychological pressure in your daily life over the past week?” Additionally, self-assessed variables including unilateral chewing, preference for hard food, sleep problems, and tinnitus were reported using binary responses (yes/no). All variables were recorded in binary format for all patients, following the methodology detailed in our previous study^6^.

(6) Ultrasonography measurements

Ultrasonography was performed using an Alpinion E-Cube 8 ultrasound system (Alpinion Medical Systems Co., Ltd., Seoul, Korea) paired with an M12L linear transducer operating at a pulse frequency range of 0–14 MHz. A fixed B-mode setting for grayscale was chosen specifically for musculoskeletal assessments. Individual adjustments for gain, focus, and depth were made for each patient. Doppler sensitivity was optimized for low flow with fixed settings (7.5 MHz Doppler frequency, pulse repetition frequency of 0.9 kHz, wall filter of 114 Hz). Ultrasound images of the major masticatory muscles were captured at specified locations (Fig. 2), allowing for the measurement of muscle thickness and cross-sectional area on both right and left sides. Measurements were performed using a program built into the Alpinion E-Cube 8 ultrasound system. Dental investigators (YHL and YHC) with relevant training conducted all ultrasound examinations.

(7) Thickness of the masticatory muscle

**Figure 1:**
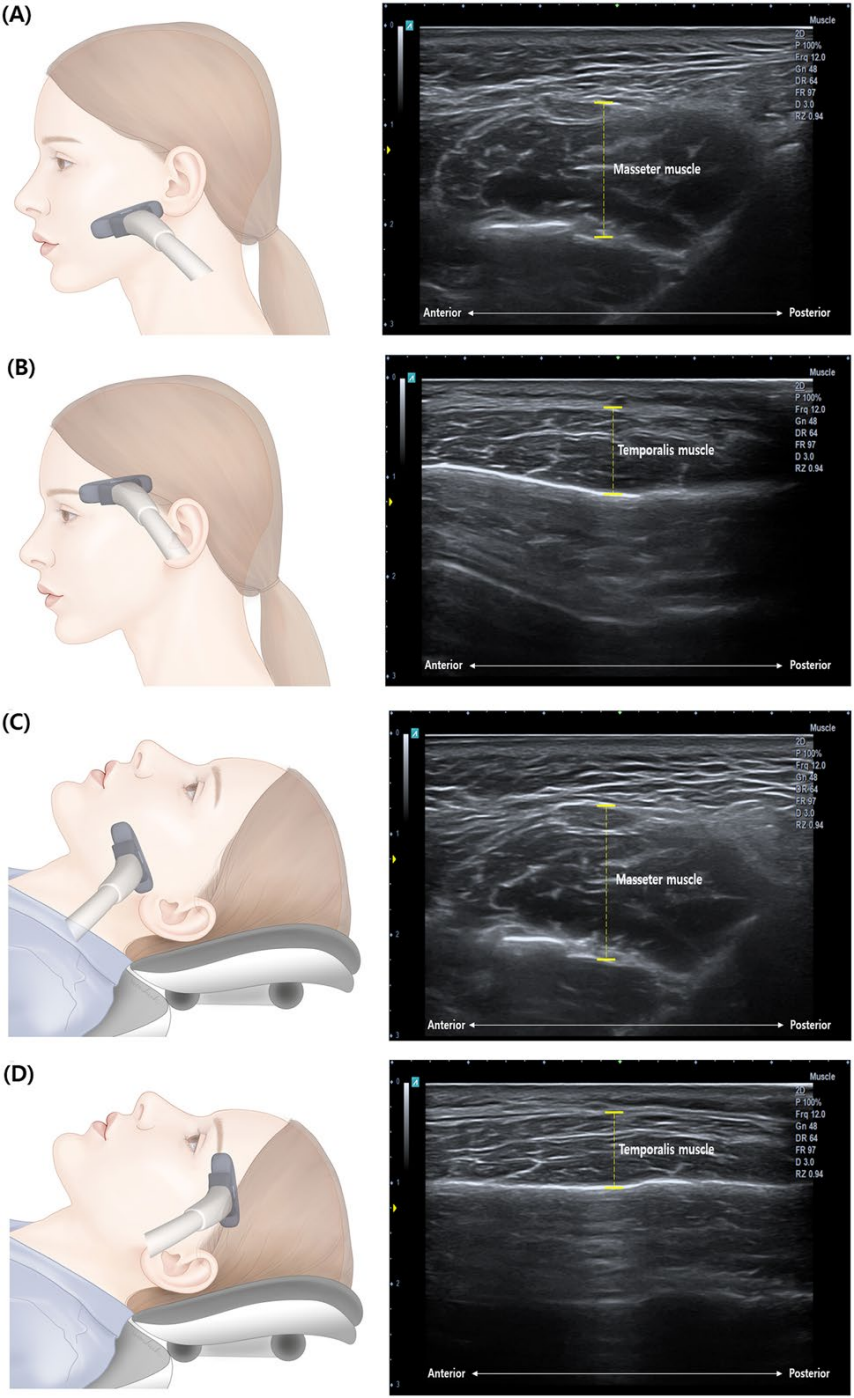
Ultrasound image paired with a schematic diagram of ultrasound observation of the main masticatory muscles—masseter and temporalis muscles. Thickness measurement of (**A**) masseter muscle in the upright position, (**B**) temporal muscle in the upright position, (**C**) masseter muscle in the supine position, and (**D**) temporal muscle in the supine position. Muscle thickness was measured sequentially in both resting and clenching conditions of the face and teeth for each position.

The masseter and temporalis muscles were initially assessed while the patient was upright and subsequently in a relaxed supine position. To start, the facial area and body were relaxed, the upper and lower lips were gently brought together, and measurements were recorded in both the resting and clenched states. Measurements commenced on the right side of the patient, where the operator was positioned, then proceeded to the left side once the right-side measurements were completed. Both the right and left sides were examined for these two masticatory muscles. Following completion of measurements in the upright posture, the same process was repeated with the patient in the supine position. This protocol aimed to determine any significant differences in muscle thickness between the upright and supine positions, as well as between the resting and clenched states. Regarding the echogenicity of major anatomical structures, normal bone interfaces appeared hyperechoic, fascia was also hyperechoic, and muscles displayed hypoechoic characteristics^43,44^. Measurement of the masseter muscle thickness was conducted at its thickest point beneath the midsection of the lower zygomatic arch. The transducer was positioned over the most prominent part of the masseter muscle, aligned parallel to the long axis of the zygomatic arch, between the lower mandibular notch and inferior border of the mandible. For the temporalis muscle, a linear probe was placed parallel to the outer edge of the eyebrow (Fig. 1). Muscle thickness was defined as the maximum distance between the outer and inner fasciae. The obtained muscle thickness in each condition for one patient represented the average of the measurements from both sides, and the values were recorded in millimeters.

(8) Reliability and measurement errors

Inter-and intra-observer reliabilities were used to assess the degree of agreement among multiple repetitions of a clinical test. All ultrasound scan parameters were measured twice by two investigators (YHL and YHC), and images were randomly presented to assess inter- and intra-observer reliability. Inter-class correlation coefficients (ICCs) were calculated, with a predefined threshold set at > 0.80 for the correlations of all items. Intra-examiner reproducibility estimated ICCs of 0.84 and 0.92, respectively. Instances where the ICC did not meet this standard value were earmarked for additional investigation and subjected to re-measurement. Disagreements were resolved through several discussions until a consensus was reached. The ICC values ranged from 0 (no reliability) to 1 (perfect reliability)^45^. Across repeated testing, the ICC consistently met the criterion (> 0.80) in all cases.

### Statistical methods

Data were analyzed using SPSS Statistics version 26.0 for Windows (IBM Corp., Armonk, NY). Continuous variables are presented as means and standard deviations (SD), and categorical variables as frequencies and percentages. The intra-rater reliability of muscle thickness and cross-sectional area measurements was assessed using the ICC coefficient, with a mean value of 0.87. Depending on the presence of bruxism in patients, they were categorized into two groups: group 1 (TMD without bruxism, TMD_nonbruxers) and 2 (TMD with bruxism, TMD_bruxers). Chi-square tests examined categorical variable distributions. Bonferroni-adjusted post-hoc analyses were used for chi-square tests to determine the equality of proportions. The thickness of masticatory muscles, including the masseter and temporalis muscles, was measured during both resting and clenching states. Differences in average thickness were investigated based on the presence or absence of bruxism and by sex using a t-test. Spearman’s correlation coefficients (r) were calculated for VAS, demographics, and ultrasonographic parameters, ranging from − 1 to + 1, with − 1 representing a perfect linear negative correlation, and + 1 representing a perfect linear positive correlation. A generalized linear model assessed factors affecting thickness changes in the masseter and temporalis muscles. The general linear model is an extension of multiple linear regression for situations involving multiple dependent variables. The extent to which the explanatory variables impacted the outcome variable was represented by the beta coefficient (B) and 95% CI values. A two-tailed *p* value of < 0.05 was considered statistically significant.

### Institutional review board approval

The research protocol adhered to the Declaration of Helsinki and received approval from the Institutional Review Board of Kyung Hee University Dental Hospital in Seoul, South Korea (KHD IRB, IRB No-KH-DT22015).

### Informed consent

Informed consent was obtained from all participants.

## Results

### Demographics

In this study, 96 patients with TMD (mean age 40.41 ± 17.88 years) diagnosed based on DC/TMD were analyzed. Among them, 30 patients (31.25%) experienced bruxism, whereas 66 (68.75%) were non-bruxers. The age difference between the TMD_nonbruxer (41.27 ± 17.92 years) and TMD_bruxer (38.51 ± 17.95 years) groups was not significant (*p* = 0.486). Females dominated the cohort with 67 (69.8%) compared with 29 males (32.2%), resulting in a total male: female ratio was 1:2.31. The female-to-male ratio was 2.47:1 for the TMD_non-bruxer group and 2.86:1 for the TMD_bruxer group.

In terms of pain characteristics, the average VAS score, reflecting pain severity, was slightly higher in the TMD_nonbruxer group (3.52 ± 1.87) than the TMD_bruxer group (2.83 ± 1.66), but not statistically significant (*p* = 0.078). Pain duration also showed no significant difference between the TMD_nonbruxer and TMD_bruxer groups (20.91 ± 36.06 vs 13.91 ± 22.19 months, *p* = 0.247). The distribution of chronicity (54.5% vs. 66.7%, *p* = 0.372) and preauricular pain (33.3% vs. 36.7%, *p* = 0.458) did not differ significantly based on the presence or absence of bruxism between the TMD_nonbruxer and TMD_bruxer groups (all *p* > 0.05) (Table 1).

**Table 1:** Demographics and TMD diagnosis based on DC/TMD among bruxers and nonbruxers.

	TMD_nonbruxers (n = 66) Mean ± SD or n (%)	TMD_bruxers (n = 30) Mean ± SD or n (%)	*p*-value
Demographics			
Age (years)^a^	41.27 ± 17.92	38.51 ± 17.95	0.486
Sex^b^			
Male	19 (28.8%)	10 (33.3%)	0.641
Female	47 (71.2%)	20 (66.7%)	
Pain characteristics			
VAS^a^	3.52 ± 1.87	2.83 ± 1.66	0.078
Pain duration (months)^a^	20.91 ± 36.06	13.91 ± 22.19	0.247
Chronicity^b^	36 (54.5%)	20 (66.7%)	0.372
Preauricular pain^b^	22 (33.3%)	11 (36.7%)	0.458
TMD diagnosis based on DC/TMD			
Arthrogenous TMD^b^	26 (41.3%)	6 (20.7%)	0.049*
Myogenous TMD^b^	15 (23.8%)	16 (53.3%)	0.003**
Mixed TMD^b^	25 (37.9%)	8 (26.7%)	0.201

^a^Results were obtained using t-tests.

^b^Results were obtained using a two-sided chi-square analysis. TMD, temporomandibular disorder; VAS, visual analog scale; Chronicity, chronic state that has elapsed more than 6 months from the onset of TMD symptoms; DC/TMD, diagnostic criteria for TMD. Statistical significance was set at *p* < 0.05. *p < 0.05. **p < 0.01. The significant values are indicated in bold font.

### Distribution of TMD subgroups according to DC/TMD

The distribution of TMD subgroups according to DC/TMD revealed a significant difference between the TMD_non-bruxer and TMD_bruxer groups. Arthrogenous TMD occurred more frequently in TMD_nonbruxers (41.3%) than in TMD_bruxers (20.7%) (*p* = 0.049). Conversely, myogenous TMD was significantly higher in TMD bruxers (53.3%) than in TMD_nonbruxers (20.7%) (*p* = 0.003). The observed frequencies of the three TMD subgroups within each group were also different in order. In the TMD_non-bruxer group, the frequency of arthrogenous TMD having the highest occurrence (41.3%), followed by mixed TMD (37.9%), and myogenous TMD (23.8%). In the TMD_bruxer group, myogenous TMD (53.3%), not arthrogenous TMD, was the most prevalent among the TMD subgroups, followed by mixed TMD (26.7%), and arthrogenous TMD (20.7%) (Table 1).

### Distribution of chief complaints and contributing factors for TMD

When examining chief complaints in patients with TMD, differences emerged between the TMD_nonbruxer and TMD_bruxer groups, particularly in limited mouth opening and muscle stiffness. Limited mouth opening (< 35 mm) was significantly more prevalent in the TMD_bruxer group than in the TMD_nonbruxer group (26.7% vs. 9.1%, *p* = 0.032). Similarly, muscle stiffness was significantly higher in TMD_bruxers than in TMD_nonbruxers (53.3% vs. 24.2%, *p* = 0.009). However, no significant differences were noted in TMJ noise, TMD pain, and occlusal dysesthesia between the two groups.

The most frequently reported chief complaint among TMD_nonbruxers was TMD pain (62.1%), followed by TMJ noise (28.8%), muscle stiffness (24.2%), occlusal dysesthesia (16.7%), and limited mouth opening (9.1%). Among TMD bruxers, the primary complaint was muscle stiffness (53.3%), followed by TMD pain (28.8%), limited mouth opening (26.7%), TMJ noise (26.7%), and occlusal dysesthesia (20.0%). In cases of TMD_nonbruxers, > 60% reported visiting the hospital due to TMD pain, while approximately 24% sought help primarily for muscle stiffness. Conversely, > 53% of TMD_bruxers complained of muscle stiffness, with TMD pain reported in approximately 28% of cases. Notably, facial muscle issues emerged as a more significant reason for TMD_bruxers to seek hospital care.

Regarding factors contributing to TMD, psychological stress showed a significantly higher prevalence in TMD_bruxers than in TMD_nonbruxers (30.3% vs. 53.3%, *p* = 0.041). Additionally, sleep issues (24.2% vs. 50.5%, *p* = 0.018) and tinnitus (12.1% vs. 40.0%, *p* = 0.003) were significantly more prevalent in TMD_bruxers than among TMD_nonbruxers. However, no significant differences were observed between the TMD non-bruxer and TMD bruxer groups in terms of unilateral chewing (34.8% vs. 40.4%, *p* = 0.653) and preference for hard food (15.2% vs. 3.3%, *p* = 0.614). Among TMD_nonbruxers, the most frequently identified contributing factor was unilateral chewing (34.8%), followed by psychological stress (30.3%), sleep issues (24.2%), preference for hard food (15.2%), and tinnitus (12.1%). Conversely, for TMD_bruxers, psychological stress (53.3%) emerged as the primary contributing factor, followed by sleep issues (50.0%), unilateral chewing, and tinnitus (40.0%), with a preference for hard food (3.3%) (Table 2).

**Table 2:** Distribution of chief complaints and contributing factors for TMD.

	TMD_nonbruxers (n = 66) Mean ± SD or n (%)	TMD_bruxers (n = 30) Mean ± SD or n (%)	*p*-value
Chief complaint			
TMJ noise	19 (28.8%)	8 (26.7%)	1.000
TMD pain	41 (62.1%)	15 (50.0%)	0.275
Limited mouth opening	6 (9.1%)	8 (26.7%)	0.032*
Muscle stiffness	16 (24.2%)	16 (53.3%)	0.009**
Occlusal dysesthesia	11 (16.7%)	6 (20.0%)	0.775
Contributing factors for TMD			
Psychological stress	20 (30.3%)	16 (53.3%)	0.041*
Unilateral chewing	23 (34.8%)	12 (40.0%)	0.653
Hard food	10 (15.2%)	1 (3.3%)	0.164
Sleep problem	16 (24.2%)	15 (50.0%)	0.018*
Tinnitus	8 (12.1%)	12 (40.0%)	0.003**

The results were obtained using a two-sided chi-square analysis. TMD, temporomandibular disorder; statistical significance was set at *p* < 0.05. *p < 0.05, **p < 0.01. The significant values are indicated in bold font.

### Factors correlated with bruxism

**Figure 2:**
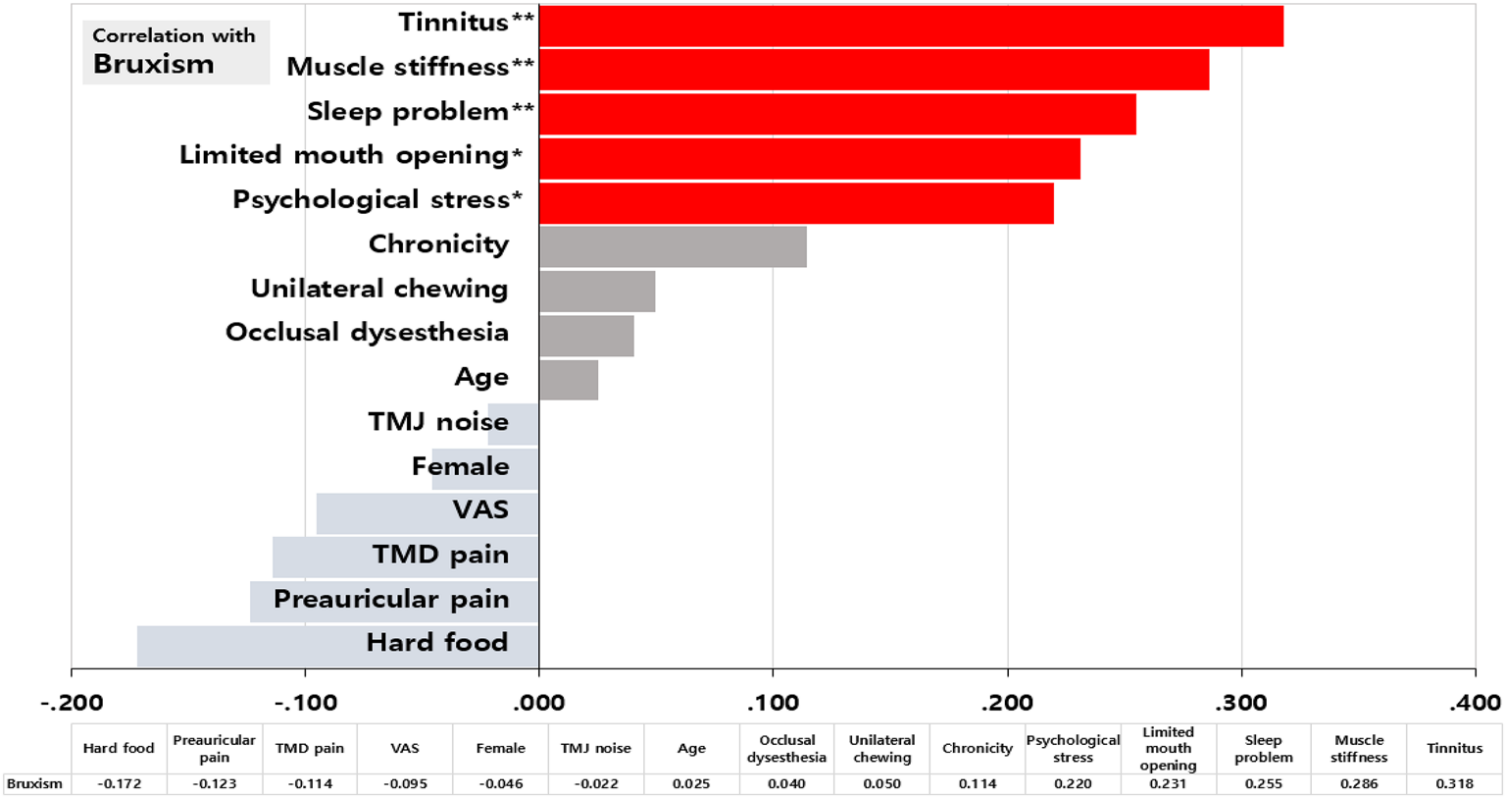
Factors correlated with bruxism (Only the factors highlighted in red displayed significant positive correlations with bruxism; no factors exhibited significant negative correlations with bruxism.) Statistical significance was set at *p* < 0.05. *p < 0.05, **p < 0.01.

When exploring clinical factors correlated with bruxism, tinnitus exhibited the strongest positive correlation among the significant clinical factors associated with bruxism (r = 0.318, *p* = 0.002). Following this, muscle stiffness (r = 0.286, *p* = 0.005), sleep problems (r = 0.255, *p* = 0.012), limited mouth opening (r = 0.231, *p* = 0.024), and psychological stress (r = 0.220, *p* = 0.031) also demonstrated noteworthy correlations However, other factors such as chronic TMD signs and symptoms, unilateral chewing, and occlusal dysesthesia did not exhibit a significant relationship with bruxism (all *p* > 0.05) (Fig. 2).

### Muscle thickness during resting and clenching

This study investigated variations in masseter and temporalis muscle thickness during resting and clenching considering patient body position. Initially, muscle thickness was measured in both the upright and supine positions. In the upright position, the masseter muscle exhibited significantly greater thickness during clenching than during rest (11.16 ± 3.03 vs 14.04 ± 3.47, *p* < 0.001), marking a difference of 2.92 ± 2.24 mm. Conversely, transitioning from rest to clenching in the upright position did not yield a substantial increase in temporal muscle thickness (7.91 ± 1.98 vs 8.39 ± 2.08, *p* = 0.103), with a marginal difference of 0.48 ± 1.21 mm.

Similarly, in the supine position, the masseter muscle exhibited significantly greater thickness during clenching than during rest (11.24 ± 2.42 vs 13.49 ± 3.09, *p* < 0.001), showcasing an average thickness difference of 2.25 ± 1.90 mm. However, in the supine position, no significant difference was observed in temporal muscle thickness between resting and clenching states (8.21 ± 2.16 vs 8.43 ± 1.94, *p* = 0.464) (Table 3).

**Table 3:** Masseter and temporal muscle thickness during resting and clenching in patients with TMD.

Total TMD patients (n = 96)		Resting Mean ± SD	Clenching Mean ± SD	*p*-value (resting vs. clenching)	Thickness difference^a^
Unit: mm					Mean ± SD
Upright position	Masseter m.	11.16 ± 3.03	14.04 ± 3.47	< 0.001***	2.92 ± 2.24
	Temporalis m.	7.91 ± 1.98	8.39 ± 2.08	0.103	0.48 ± 1.21
Supine position	Masseter m.	11.24 ± 2.42	13.49 ± 3.09	< 0.001***	2.25 ± 1.90
	Temporalis m.	8.21 ± 2.16	8.43 ± 1.94	0.464	0.22 ± 0.76

The results were obtained using t-tests. TMD, temporomandibular disorder; m, masseter muscle.

^a^Difference between muscle thickness at clenching and that at rest. Statistical significance was set at *p* < 0.05. *p < 0.05, **p < 0.01, ***p < 0.001. The significant values are indicated in bold font.

### Muscles thickness of TMD_bruxers in upright position

**Figure 3:**
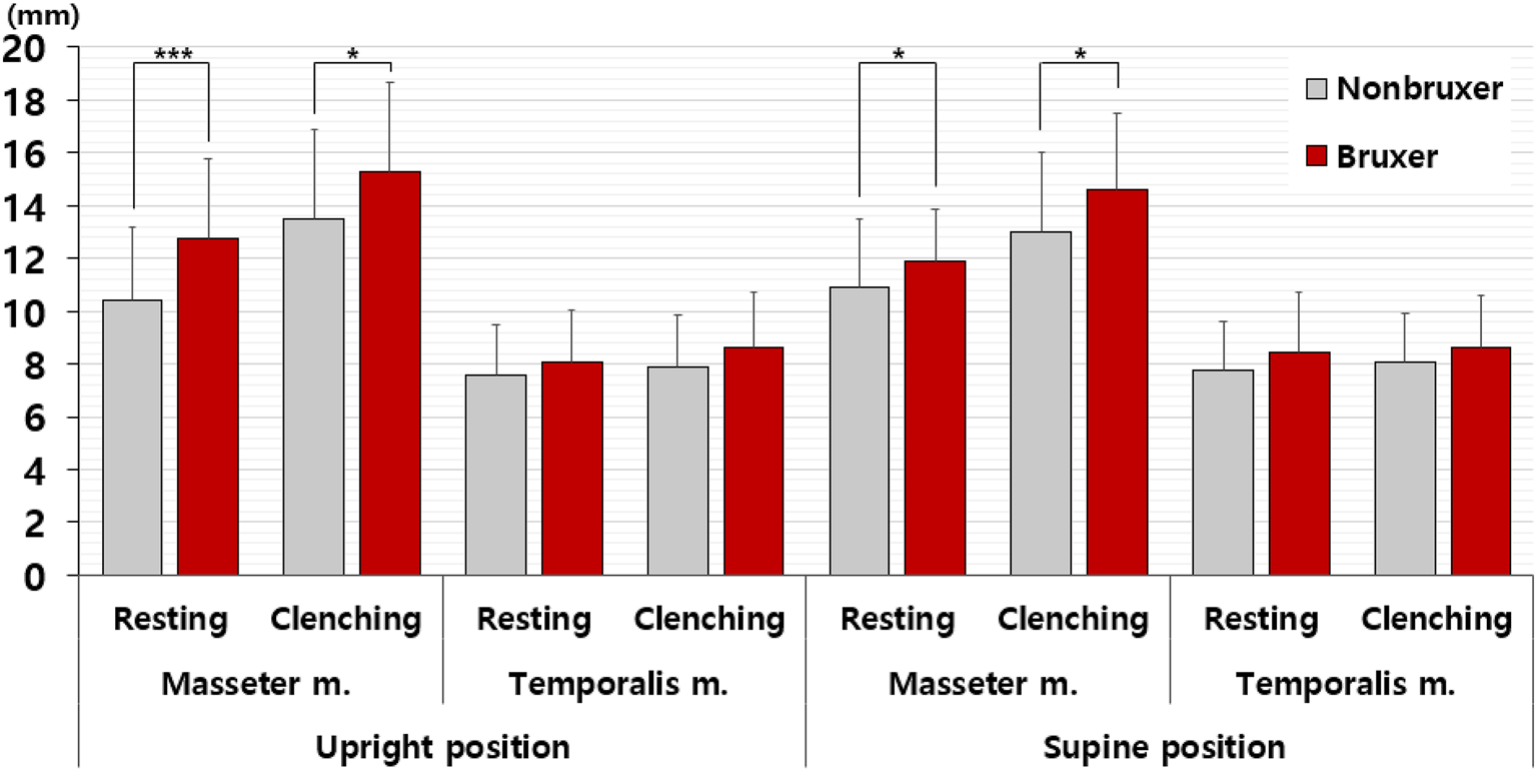
Differences in muscle thickness between bruxers and non-bruxers among patients with TMD.

Comparing muscle thickness between TMD_nonbruxers and TMD_bruxers in both upright and supine positions revealed that only the masseter muscle exhibited a significant increase in thickness during clenching compared with resting (Fig. 3). The study measured the thicknesses of the masseter and temporal muscles, key masticatory muscles, in resting and clenching states for both TMD_non-bruxers and TMD_bruxers in the upright and supine positions. In all cases, the masticatory muscle thickness was greater in TMD bruxers compared with TMD non-bruxers, particularly during clenching rather than at rest. However, the differences in muscle thickness between the two conditions were significant only in the masseter muscle, not in the temporalis muscle.

A significant disparity in masticatory muscle thickness was observed in the masseter muscle between TMD_non-bruxers and TMD_bruxers. In the upright position, the masseter muscle was significantly thicker in TMD bruxers than in TMD_nonbruxers during resting (10.44 ± 2.77 mm vs. 12.73 ± 3.03 mm, *p* < 0.001). Furthermore, during clenching in the upright position, the masseter muscle thickness in TMD bruxers increased significantly compared with TMD non-bruxers (13.48 ± 3.41 mm vs 15.26 ± 3.39 mm, *p* = 0.021). Regarding the temporal muscles, no significant differences were observed in muscle thickness between TMD_nonbruxers and TMD_bruxers during resting (7.59 ± 1.90 vs 8.05 ± 2.01 mm, *p* = 0.284) or clenching (7.89 ± 1.96 mm vs 8.61 ± 2.12 mm, *p* = 0.114) in the upright position (Table 4).

**Table 4:** Ultrasonography examination of masticatory muscles in upright and supine positions.

Unit: mm			TMD_nonbruxers (n = 66) Mean ± SD	TMD_bruxers (n = 30) Mean ± SD	*p*-value (nonbruxers vs. bruxers)	Male (n = 29)	Female (n = 67)	*p*-value (male vs. female)
						Mean ± SD	Mean ± SD	
Upright position	Masseter m.	Resting	10.44 ± 2.77	12.73 ± 3.03	< 0.001***	12.46 ± 3.14	10.59 ± 2.83	0.008**
		Clenching	13.48 ± 3.41	15.26 ± 3.39	0.021*	14.98 ± 3.00	13.69 ± 3.59	0.074
	Temporal m.	Resting	7.59 ± 1.90	8.05 ± 2.01	0.284	7.97 ± 1.75	7.76 ± 2.45	0.688
		Clenching	7.89 ± 1.96	8.61 ± 2.12	0.114	8.49 ± 1.83	8.16 ± 2.61	0.540
Supine position	Masseter m.	Resting	10.93 ± 2.55	11.92 ± 1.97	0.043*	12.43 ± 2.74	10.72 ± 2.08	0.004**
		Clenching	12.97 ± 3.05	14.62 ± 2.90	0.014*	15.23 ± 3.35	12.73 ± 2.66	< 0.001***
	Temporal m.	Resting	7.74 ± 1.85	8.43 ± 2.27	0.119	8.44 ± 2.94	8.11 ± 1.58	0.578
		Clenching	8.04 ± 1.86	8.61 ± 1.96	0.179	8.53 ± 2.37	8.39 ± 1.74	0.765

The results were obtained using t-tests. m, masseter muscle. Statistical significance was set at *p* < 0.05. *p < 0.05, **p < 0.01, ***p < 0.001. The significant values are indicated in bold font.

### Muscle thickness of TMD_bruxer in supine position

To discern positional trends, the procedures conducted in the upright position were replicated in the supine position. The observed tendency in the upright position mirrored that observed in the supine position. Even in the supine position, a significant difference in muscle thickness between TMD_nonbruxers and TMD_bruxers was evident solely in the masseter muscles. During supine position at rest, the masseter muscle was significantly thicker in TMD_ bruxers than in TMD_nonbruxers (10.93 ± 2.55 vs. 11.92 ± 1.97 mm, *p* = 0.043). When clenching in the supine position, the masseter muscle thickness significantly increased in TMD_bruxers compared with TMD_nonbruxers (12.97 ± 3.05 vs 14.62 ± 2.90 mm, *p* = 0.014). This implies that patients with TMD with bruxism exhibited thicker masseter muscles in the supine position, both at rest and during clenching, compared with those without bruxism. Regarding the temporalis muscles, no statistically significant difference in muscle thickness between TMD_nonbruxers and TMD_bruxers during resting (7.74 ± 1.85 vs 8.43 ± 2.27 mm, *p* = 0.284) or clenching (8.04 ± 1.86 mm vs 8.61 ± 1.96 mm, *p* = 0.114) in the supine position.

​When comparing the muscle thickness of the temporalis muscle between TMD.

_nonbruxers and TMD_bruxers in the supine position, no significant difference was found during resting (7.74 ± 1.85 vs 8.43 ± 2.27 mm, *p* = 0.284) or clenching (8.04 ± 1.86 mm vs 8.61 ± 1.96 mm, *p* = 0.114). In other words, no discernable difference existed in the thickness of the temporalis muscle between the TMD_nonbruxers and TMD_bruxers either at rest or during clenching in the supine position (Table 4).

### Sex-differences in muscle thickness

**Figure 4:**
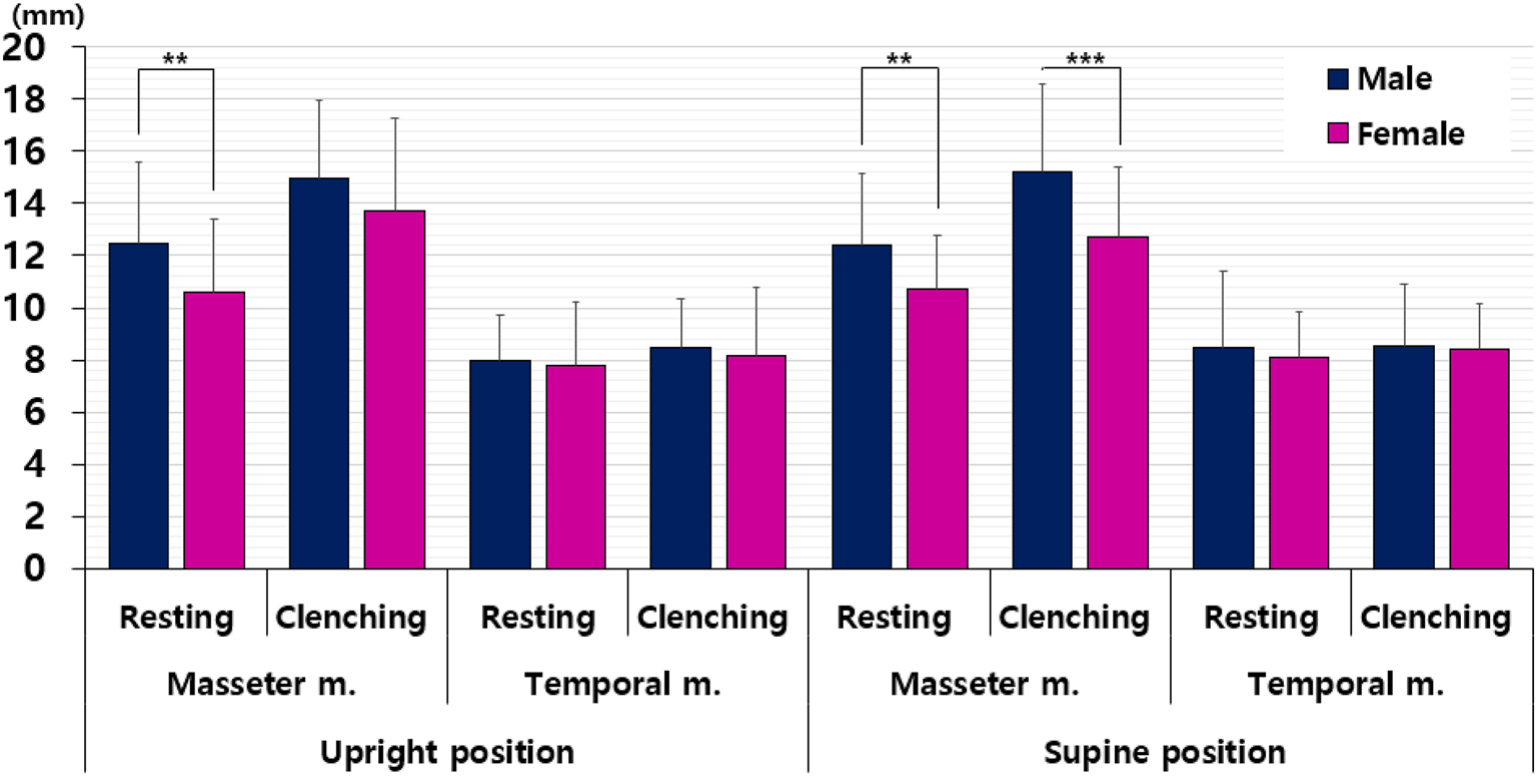
Differences in muscle thickness between sexes in patients with TMD.

This study investigated differences in masseter and temporalis muscle thickness between males and females across various body positions, specifically comparing muscle states during rest and clenching. Males generally exhibited greater thickness in both the masseter and temporalis muscles than females, with statistical significance observed primarily in the masseter muscle. When assessing muscle thickness based on sex, in the upright position, the masseter muscle was notably thicker in males than in females only at rest. However, in the supine position, the masseter muscle was significantly thicker in men than in women, both at rest and in the supine position (all *p* < 0.05). Conversely, no significant difference in thickness between sexes was noted in the temporalis muscle, irrespective of body position and resting/clenching conditions (Fig. 4).

Specifically, in the upright resting position, males exhibited significantly thicker masseter muscles compared with females (12.46 ± 3.14 vs 10.59 ± 2.83 mm, *p* = 0.008). Interestingly, in the supine position, no significant mean differences were observed in masseter and temporalis muscle thicknesses between sexes. During both resting (12.43 ± 2.74 vs 10.72 ± 2.08, *p* = 0.004) and clenching (15.23 ± 3.35 vs 12.73 ± 2.66, *p* < 0.001) in the upright position, males exhibited significantly thicker masseter muscles than females, indicating substantial distinctions. However, no sex-based differences in temporalis muscle thickness were observed during both resting and clenching, irrespective of body position.

In the upright position, temporalis muscle thickness exhibited no statistically significant difference between males and females during both resting (7.97 ± 1.75 vs 7.76 ± 2.45 mm, *p* = 0.688) and clenching (8.49 ± 1.83 vs 8.16 ± 2.61 mm, *p* = 0.540). Similarly, in the supine position, differences in temporal muscle thickness between males and females during resting (8.44 ± 2.94 vs 8.11 ± 1.58, *p* = 0.578) and clenching (8.53 ± 2.37 vs 8.39 ± 1.74, *p* = 0.765) were not statistically significant (Table 4).

### Comparison of muscle thickness between TMD subgroups

Based on the DC/TMD classification, patients were categorized into arthrogenous TMD (n = 32), myogenous TMD (n = 31), and mixed TMD (n = 33) groups. Analysis of masseter and temporalis muscle thickness, considering patient posture and resting versus clenching statuses, revealed no statistically significant differences. Across the three TMD subgroups (arthrogenous, myogenous, and mixed), both masseter and temporal muscles tended to be thicker during clenching than during the resting state. However, these differences did not reach statistical significance (Table 5).

**Table 5:** Muscle thickness differences between TMD subgroups based on DC/TMD.

Unit: mm			Arthrogenous TMD (n = 32) Mean ± SD	Myogenous TMD (n = 31) Mean ± SD	Mixed TMD (n = 33) Mean ± SD	*p*-value
Upright position	Masseter m.	Resting	11.03 ± 32	12.02 ± 2.81	10.47 ± 3.05	0.122
		Clenching	13.96 ± 3.74	14.88 ± 3.27	13.33 ± 3.37	0.207
	Temporal m.	Resting	7.38 ± 1.87	8.37 ± 2.26	7.98 ± 1.71	0.135
		Clenching	7.90 ± 1.99	8.81 ± 2.38	8.47 ± 1.84	0.220
Supine position	Masseter m.	Resting	11.29 ± 1.85	11.89 ± 2.07	10.59 ± 3.03	0.098
		Clenching	13.14 ± 2.83	14.06 ± 2.84	13.28 ± 3.54	0.454
	Temporal m.	Resting	7.96 ± 2.54	8.58 ± 2.20	8.11 ± 1.72	0.505
		Clenching	8.03 ± 1.83	8.89 ± 2.21	8.38 ± 1.72	0.204

The results were obtained using t-tests. TMD, temporomandibular disorder; m, masseter muscle; statistical significance was set at *p* < 0.05. There was no significant difference in muscle thickness between the TMD subgroups (all *p* > 0.05).

### Correlation coefficient (r) between thicknesses of masseter and temporal muscles

The correlation between masseter and temporalis muscle thickness, considering patient posture and resting versus clenching states, was investigated. Interestingly, a very strong correlation (correlation coefficient *r* > 0.9) was found between muscle thickness during resting and clenching states. This suggests that in patients with TMD, thicker muscles observed during the resting period corresponded proportionally to thicker muscles during clenching.

In TMD_nonbruxers, during clenching in the upright position, a significantly strong correlation was found between the masseter muscle thickness and its resting state in the upright position (r = 0.699, *p* < 0.001). Similarly, a strong correlation was observed between the resting-state thickness of the temporalis muscle in the upright position and its thickness during clenching in the same position (r = 0.865, *p* < 0.001). Notably, in the supine position among TMD_non-bruxers, the resting-state thickness of the temporalis muscle exhibited the strongest positive correlation with its thickness during clenching in the same position (r = 0.922, *p* < 0.001).

**Figure 5:**
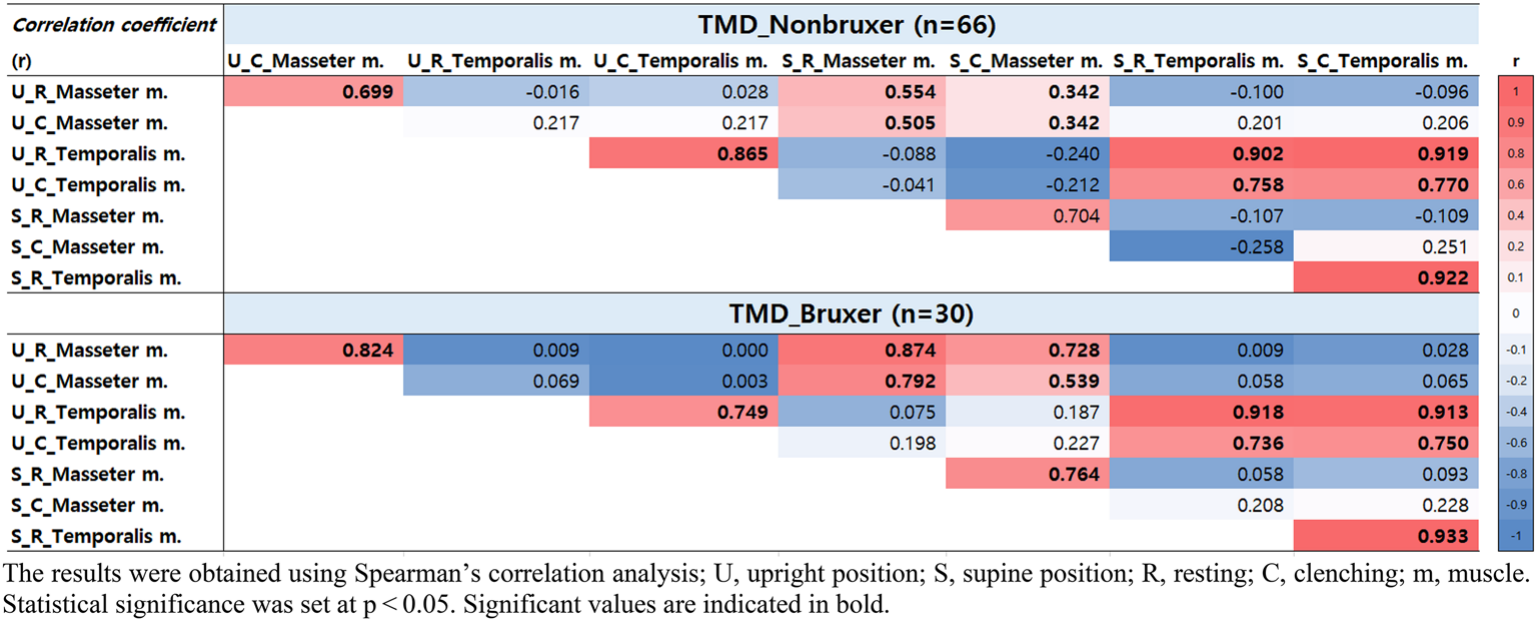
Correlation of thicknesses in each condition of masseter and temporal muscles. The results were obtained using Spearman’s correlation analysis; U, upright position; S, supine position; R, resting; C, clenching; m, muscle. Statistical significance was set at *p* < 0.05. Significant values are indicated in bold.

In TMD_bruxers, during clenching in the upright position, a significantly strong correlation was observed between masseter muscle thickness and its resting state in the upright position (r = 0.824, *p* < 0.001). Similarly, a strong correlation was observed between the resting-state thickness of the temporalis muscle in the upright position and its thickness during clenching in the same position (r = 0.749, *p* < 0.001). Interestingly, in the supine position among TMD_bruxers, the resting-state thickness of the temporalis muscle showed the strongest positive correlation with its thickness during clenching in the same position (r = 0.933, *p* < 0.001) (Fig. 5).

### Generalized linear model for masticatory muscle thickness changes

A generalized linear model was employed to investigate the factors influencing masseter and temporalis muscle thicknesses in the resting position while upright, aiming to quantify their respective effects. All previously examined clinical and ultrasonographic factors were used as explanatory variables, with masseter and temporalis muscle thicknesses as the outcome variables, enabling the creation of individual generalized linear models for each muscle.

Concerning masseter muscle thickness, being female, in comparison to males, emerged as a predictive factor associated with reduced masseter muscle thickness (B = − 1.018, 95% CI − 1.855 to − 0.181, *p* = 0.017). Interestingly, bruxism significantly contributed to increased masseter muscle thickness (B = 0.868, 95% CI 0.567 to 1.169, *p* = 0.048). However, besides sex and bruxism, none of the clinical factors showed significant predictive power for masseter muscle thickness.

Three factors significantly contributed to temporal muscle thickness. Female sex was identified as a predictor associated with decreased temporal muscle thickness (B = − 0.201, 95% CI − 0.299 to − 0.103, *p* = 0.011). Moreover, increased age was a significant predictor of temporal muscle decline (B = − 0.003, 95% CI − 0.005 to 0.000, *p* = 0.038). Additionally, muscle stiffness emerged as a predictor of decreased temporal muscle thickness (B = − 1.373, 95% CI − 2.369 to − 0.376, *p* = 0.007).

Furthermore, several clinical factors, including TMJ noise, TMD pain, tinnitus, sleep issues, occlusal dysesthesia, and TMD subgroups diagnosed using DC/TMD, did not exhibit significant explanatory power for the thickness of these two muscles (Table 6).

**Table 6:** Generalized linear model for thickness changes of masseter and temporal muscles.

Total TMD patients (n = 96)	Upright position_Resting_Masseter m					Upright position_Resting_Temporalis m				
	Coefficient (B)	Standard error	95% wald CI_lower	95% wald CI_upper	*p*-value	Coefficient (B)	Standard error	95% wald CI_lower	95% wald CI_upper	*p*-value
Demographics										
Age	0.001	0.010	− 0.019	0.022	0.891	**− 0.003**	0.001	− 0.005	0.000	0.038*
Female [ref. = male]	− 1.018	0.427	− 1.855	− 0.181	0.017*	**− 0.201**	0.496	− 0.299	− 0.103	0.011*
Clinical characteristics										
VAS [ref. < average value]	− 0.028	0.407	− 0.825	0.769	0.945	0.034	0.051	− 0.066	0.134	0.500
Preauricular pain [ref. = none]	− 0.006	0.006	− 0.018	0.005	0.277	− 0.001	0.001	− 0.003	0.000	0.073
Chronicity [ref. = none]	− 0.028	0.403	− 0.818	0.762	0.945	− 0.018	0.051	− 0.117	0.081	0.720
TMJ noise [ref. = none]	− 0.558	0.401	− 1.343	0.227	0.164	0.006	0.050	− 0.093	0.104	0.908
TMD pain [ref. = none]	− 0.293	0.374	− 1.027	0.440	0.433	− 0.053	0.047	− 0.145	0.038	0.256
Locking [ref. = none]	0.456	0.560	− 0.642	1.554	0.416	1.098	0.745	− 0.362	2.558	0.140
Stiffness [ref. = none]	0.488	0.385	− 0.266	1.243	0.205	**− 1.373**	0.508	− 2.369	− 0.376	0.007**
Contributing factor										
Bruxism [ref. = none]	0.868	0.443	0.567	1.169	0.048*	0.010	0.056	− 0.099	0.119	0.862
Psychological stress [ref. = none]	0.127	0.394	− 0.646	0.900	0.748	− 0.089	0.049	− 0.184	0.007	0.069
Unilateral chewing [ref. = none]	− 0.336	0.365	− 1.052	0.380	0.358	0.038	0.046	− 0.052	0.127	0.411
Hard food [ref. = none]	0.020	0.536	− 1.030	1.070	0.970	− 0.534	0.710	− 1.926	0.858	0.452
Sleep problem [ref. = none]	0.601	0.397	− 0.177	1.379	0.130	− 0.012	0.012	− 0.056	0.033	0.091
Tinnitus [ref. = none]	− 0.121	0.438	− 0.979	0.736	0.782	0.011	0.055	− 0.097	0.119	0.843
Occlusal dysesthesia [ref. = none]	0.279	0.438	− 0.579	1.137	0.524	0.019	0.055	− 0.089	0.127	0.727
TMD diagnosis based on DC/TMD										
Mixed TMD [ref. = arthrogenous TMD]	− 0.002	0.459	− 0.901	0.897	0.996	0.149	0.126	− 0.098	0.395	0.237
Myogenous TMD [ref. = arthrogenous TMD]	0.189	0.462	− 0.717	1.096	0.682	0.085	0.127	− 0.163	0.333	0.502

The results were obtained using a generalized linear model.

*CI* confidence interval, *m* muscle, *DC/TMD* diagnostic criterion for temporomandibular disorders.

Significant values are in bold.

*p-value < 0.05.

**p-value < 0.01.

## Discussion

We measured masticatory muscle thickness in both upright (seated in a unit chair) and supine positions to assess its potential variation due to body position. Comparative analyses evaluated muscle thickness differences between these positions. Additionally, we examined muscle thickness changes between resting and clenching states within each body position. We measured masseter and temporal muscle thickness in TMD_nonbruxer and TMD_bruxer groups, both at rest and during clenching in upright and supine positions. Overall, masticatory muscle thickness increased in TMD bruxers compared with TMD non-bruxers, predominantly during clenching rather than at resting. However, significant differences were found only in the masseter muscle, not the temporalis muscle.

The masseter muscle is of paramount importance during mastication, playing the most crucial role among the masticatory muscles. Electromyography studies revealed that the masseter muscle was more significantly affected than the temporalis muscle under maximal clenching or mastication^32,33^. Interestingly, in children, masseter muscle thickness had a positive correlation with bite force, while the anterior temporalis thickness was not associated with bite force^46^. The thickness of the masseter muscle in healthy individuals was significantly increased during contraction compared to at rest (from 1.09 to 1.40 cm), while the increase in the temporalis muscle was not statistically significant (from 0.88 to 0.98 cm)^34^. However, in adults aged 18–45 years, the differences in masseter and temporalis muscle thickness based on the presence or absence of bruxism during rest and dental clenching were not statistically significant^47^. In this study, notable differences in the masseter muscle were observed in TMD patients based on clenching and body posture. Mastication requires coordinated efforts and balance among various masticatory muscles. Given that the masseter muscle possesses larger muscle thickness, contributes more significantly to bite force, and plays a more crucial role in mastication compared to the temporalis muscle, along with anatomophysiological differences, conversely, the masseter muscle may be more susceptible to the influence of oral function/parafunction.

Bruxism defined as involuntary and excessive teeth grinding, involves forceful contact between upper (maxillary) and lower (mandibular) teeth surfaces. In 2013, an expert panel defined it as repetitive masticatory muscle activity^48^. This activity, termed bruxism, involves teeth clenching or grinding, accompanied by mandible straightening or pushing. It can occur during sleep (sleep grinding) or while awake (awake grinding)^12,48^. Bruxism involves involuntary and repetitive movements of the mandible muscles, with studies more frequently investigating its association with the masseter muscle than with the temporalis muscle among the main masticatory muscles^49^. Bruxism increases the risk of oral complications, including tooth wear, pain, and discomfort in the masticatory muscles and TMJ^50^. However, noting that not all cases of bruxism necessarily lead to these complications is important. When examining the EMG activity in the masseter and temporalis muscles, research revealed lower masseter EMG activity in patients with TMD than in healthy controls, whereas anterior temporalis EMG activity showed no difference between the two groups^51^. Palinkas et al. found no statistically significant differences in muscle thickness and EMG activity of the masseter and temporalis muscles based on the presence or absence of SB^47^. Further investigation is crucial to clarify whether the masseter muscle is more impacted than the temporalis muscle in patients with TMD or if the masseter muscle plays a more proactive role in bruxism than the temporalis muscle.

Sex-based differences in muscle thickness were evident solely in the masseter muscle, not the temporalis muscle. Among the four primary masticatory muscles, the masseter exerts the greatest force^6,52^. When comparing masseter and temporal muscle thickness between females and males based on body position and muscle status (resting or clenching), males displayed larger thickness in both muscles than females, with statistical significance observed only in the masseter muscle. Variations in skeletal muscle among sexes encompassed differences in muscle fiber count, Type I and II fiber distribution, although certain variances were specific to particular muscles^53^. An earlier EMG study reported higher masticatory activity in males than in females^51^. Specifically, in this study, masseter thickness during rest was measured at 12.46 ± 3.14 mm in males and 10.59 ± 2.83 mm in females. In a separate examination involving healthy Korean adults, ultrasonography recorded masseter muscle thickness at 11.3 ± 1.2 mm in males and 9.8 ± 1.3 mm in females^54^. These discrepancies likely stem from variations in age, TMD presence, measurement sites, and device types used. Given that this research is the first to compare temporalis muscle thickness in TMD_bruxers and nonbruxers, additional investigation was conducted to determine whether sex, body position, and bruxism have a lesser impact on the temporalis muscle in TMD patients than the masseter muscle.

Tinnitus, muscle stiffness, sleep issues, psychological stress, restricted mouth opening, and bruxism show a positive correlation in patients with TMD. First, tinnitus might be linked to TMD, with patients reporting symptoms such as ear fullness and pain, dizziness, hearing loss, and tinnitus. The prevalence of ear-related symptoms reaches 87% among patients with TMD, irrespective of sex or age^55^. Although not focused on patients with TMD, another study found a higher frequency of tinnitus in patients with SB and chronic facial pain^56^. Buergers et al. discovered that tinnitus is eight times more prevalent in patients with TMD than in those without TMD^57^, strongly suggesting a robust association between tinnitus and TMD. To the best of our knowledge, this study was the first to directly investigate the correlation coefficient between tinnitus and bruxism. Moreover, muscle stiffness and bruxism exhibited a significant and positive correlation. A comprehensive review highlighted that bruxism can trigger facial pain originating from the muscles^58^. The presence of self-reported bruxism was correlated with anxiety, somatization, neuroticism, and psychological stress^59,60^, suggesting that bruxism may function as the body's response or coping strategy to psychological problems.

A recent review highlighted bruxism as a factor contributing to various health issues, including tooth wear, increased tooth mobility, fractures, sensitivity, inflamed and receding gums, and tongue deformation. Bruxism is linked with TMD, causing limitations in mouth opening, orofacial muscle pain, headaches, earaches, shoulder stiffness, disrupted sleep, and disturbances for bedpartners due to associated noises^61^. Stress is defined as an emotional experience wherein the perceived demands exceed the available resources. A systematic review and meta-analysis, involving three articles, found that individuals under stress were more prone to exhibiting bruxism compared with healthy individuals^21^. Moreover, somatic anxiety has been linked to SB^62^. However, there exists an alternate perspective suggesting that bruxism might act as an adaptive coping mechanism against psychological stress^59^. Although bruxism is not a specific disorder on its own^50^, its co-occurrence with TMD can exacerbate symptoms, underscoring the need for ongoing, comprehensive exploration of bruxism.

Ultrasonography emerges as a promising diagnostic tool for TMD, offering advantages over MRI, the current gold standard for TMJ assessment. Unlike CBCT, optimized for bone structures, ultrasonography is cost-effective and widely available in outpatient clinics^63^. The test is non-invasive, quick (typically 10–15 min), and well-tolerated even by diverse age groups. The protocol for ultrasonography entails no radiation or associated risks, while being painless. It allows dynamic real-time assessment with the mouth open or closed and facilitates direct communication with the patient to focus on painful areas. Importantly, sedation is not necessary for children or older adults^64^. Furthermore, Ariji et al. compared ultrasound examination and MRI in patients, highlighting their potential for diagnosing or treating masticatory muscle pain^65^. Muscle thickness investigation has traditionally been straightforward^66–68^. Given the advantages of ultrasonography and its nascent stage of implementation in TMD, more reliable studies focusing on the anatomical and pathological traits of the TMJ and masticatory muscle are required. Although this study demonstrated high agreement among examiners in measuring muscle thickness, its ability to identify active inflammation and arthritic changes as accurately as MRI or CBCT remains uncertain. Furthermore, developing and establishing protocols for easier and quicker detection of TMJ effusion, arthritis, and myalgia in patients with TMD is necessary.

This study has several limitations. First, bruxism was not diagnosed using polysomnography but relied on patient self-reports and a questionnaire. Despite numerous prior studies relying on self-reported bruxism^69–71^, this approach may pose a significant limitation in affirming the robustness of the research. Furthermore, muscle thickness was not investigated by segregating patients with TMD based on signs and symptoms on affected versus non-affected sides. Instead, for research purposes, we averaged the right and left muscle thickness. Consequently, comparisons between muscle values from the TMD patient group and healthy controls were not feasible. Among masticatory muscles, the lateral and medial pterygoid muscles have limited accessibility to ultrasound probes. Nevertheless, considering the involvement of the superior head of the lateral pterygoid muscle in clenching and the role of the medial pterygoid muscle in mandibular elevation and mastication^72–75^, further research on these muscles is also warranted. Nevertheless, this study is pioneering in directly comparing masseter and temporalis muscle thickness in TMD_bruxers and TMD_nonbruxers, considering patient posture and clenching condition.

## Conclusions

In this study, we aimed to assess ultrasonography-based changes in the thickness of major masticatory muscles—masseter and temporalis—in various body positions and during occlusal resting and clenching. Clenching led to increased masseter muscle thickness compared with that at resting, with bruxism further contributing to this increase. However, the temporalis muscle thickness showed no significant change owing to these factors. Posture variations only affected the masseter muscle thickness, not the temporalis. In patients with TMD, bruxism showed a positive correlation with tinnitus, muscle stiffness, sleep issues, psychological stress, and limited mouth opening. To enhance understanding, longitudinal studies tracking masticatory muscle thickness changes over time, considering clinical factors, are warranted, preferably with a multicenter approach.

## Data Availability

The datasets used and/or analyzed during the current study are available from the corresponding author upon reasonable request.
